# Identifying pathways to metal–organic framework collapse during solvent activation with molecular simulations†

**DOI:** 10.1039/d3ta04647h

**Published:** 2023-11-09

**Authors:** Joseph R. H. Manning, Gaël Donval, Mat Tolladay, Tom L. Underwood, Stephen C. Parker, Tina Düren

**Affiliations:** a Centre for Integrated Materials, Processes and Structures, Department of Chemical Engineering, University of Bath UK T.Duren@bath.ac.uk; b Department of Chemistry, University College London UK; c Department of Chemical Engineering, University of Manchester UK Joseph.Manning@manchester.ac.uk; d Department of Chemistry, University of Bath UK

## Abstract

Metal–organic framework (MOF) materials are a vast family of nanoporous solids with potential applications ranging from drug delivery to environmental remediation. Application of MOFs in these scenarios is hindered, however, by difficulties in MOF ‘activation’ after initial synthesis – removal of the synthesis solvent from the pores to make the pore space accessible – often leading to framework collapse if improperly performed. While experimental studies have correlated collapse to specific solvent properties and conditions, the mechanism of activation-collapse is currently unknown. Developing this understanding would enable researchers to create better activation protocols for MOFs, accelerating discovery and process intensification. To achieve this goal, we simulated solvent removal using grand-canonical Monte Carlo and free energy perturbation methods. By framing activation as a fluid desorption problem, we investigated activation processes in the isoreticular metal organic framework (IRMOF) family of MOFs for different solvents. We identified two pathways for solvent activation – the solvent either desorbs uniformly from each individual pore or forms coexisting phases during desorption. These mesophases in turn lead to large capillary stresses within the framework, corroborating experimental hypotheses for the cause of activation-collapse. Finally, we found that the activation energy of solvent removal increased with pore size and connectivity due to the increased stability of solvent mesophases, matching experimental findings. Using these simulations, it is possible to screen MOF activation procedures, enabling rapid identification of ideal solvents and conditions and thus enabling faster development of MOFs for practical applications.

## Abstract 2

Visualisation of MOF activation mechanisms, showing gas-like solvent behaviour leading to successful activation (top), and capillary-like solvent behaviour leading to collapse (bottom).

## Graphical abstract

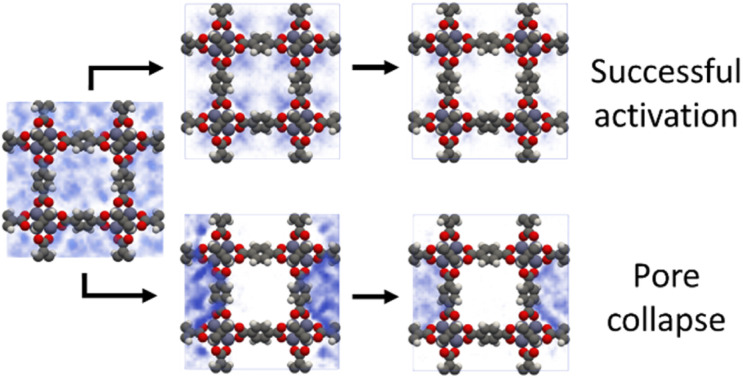

## Introduction

Metal–organic frameworks (MOFs) are a class of porous crystalline materials which have shown potential for a range of applications.^1^ Their structure, consisting of organic linkers connecting clusters of metal atoms, creates a phase-space with unparalleled diversity – over 100 000 MOFs have been experimentally synthesized to date,^2–5^ and several million more hypothetical structures have been identified.^6–10^ MOFs are the subject of intense research due to their record-breaking levels of porosity,^11–15^ modular construction enabling fine-tuning of metal^16–18^ and linker^19–22^ chemistry, and the resultant diversity in terms of materials properties.^2,23–27^ Accordingly, there have been many studies focusing on the applicability of MOFs to industrially important small molecule separation^1,28–30^ and storage^31–34^ applications.

A key challenge in synthesizing MOF materials lies in their ‘activation’ after synthesis, a step which removes solvent adsorbed within the MOF's pores during the synthesis procedure.^35–37^ Activation is essential to open up the surface area of MOF materials, a requirement for the vast majority of their applications. However, improper activation can easily lead to the pore structure becoming inaccessible, rendering the resultant materials useless.^38^ Detailed experimental studies have identified a range of plausible causes for poor activation including incomplete guest removal, surface blocking of the accessible pores, and total or partial collapse of the crystalline phases due to framework degradation.^39^ Further, seemingly minor changes to a framework structure can drastically alter their performance during activation due to subtle changes in the metal–linker chemistry^40^ or framework mechanical stability.^41,42^ Attempts to improve activation methods to increase the reliability of MOF materials are therefore hindered by incomplete understanding and characterisation of these phenomena. This is particularly true of framework collapse, which is usually only identified by the loss of sample crystallinity upon activation.^39^

Activation generally is done by heating the MOF material under reduced pressure in order to remove the confined fluid.^35^ Direct MOF activation from the synthesis solvent – usually a polar, high boiling point compound such as dimethylformamide (DMF) – is rarely performed both due to the harsh conditions required and tendency to trigger activation-collapse. Instead, the reaction solvent is often exchanged for a more volatile alternative.^35,43,44^ Solvent exchange is not universally effective, however, as the activation behaviour of specific MOF–solvent pairs cannot be easily predicted,^41^ and some MOFs even collapse upon solvent exchange.^45^ Therefore, laborious trial-and-error development of activation methods are required for each new material developed.^41^ Furthermore, incorporation of solvent exchange to MOF production methods adds a further processing step, with its own requirements for validation and optimisation.^44^ As a result of these difficulties, investigation into the how^39^ and why^41,46^ of activation-collapse are essential to overcome the barriers towards cost-effective MOF scaleup.

Experimental studies into MOF activation have provided several rules of thumb for successful solvent activation, providing indicators of the underlying phenomena controlling activation-collapse. Lower surface tension and more volatile solvents such as acetone,^41^ dichloromethane,^44^ and hexane^39^ are recommended over higher surface tension, less volatile alternatives (Tables 1 and S1†).^37^ In terms of the framework components, overly long and rigid linkers are discouraged,^41^ as they may enable larger solvent phases to form with greater associated capillary stress. Similarly, twisted^41^ or flexibile^47^ linkers can lead to mechanical torsion in the metal–ligand bonds, thereby reducing the capillary stress required to break metal–ligand bonds. Beyond these rules of thumb, some MOFs require further considerations to prevent adverse outcomes of activation. For example, MOFs containing coordinatively unsaturated metal sites require solvent–metal coordination to be broken in order to activate the open metal sites,^48^ and flexible MOFs can behave differently after activation depending on both crystal size and the activation solvent used.^49^

**Table 1:** Surface tension and boiling point values for some commonly used solvents in the synthesis and activation of MOFs. Values taken from ref. 37

Solvent	Bulk surface tension (mN m^−1^)	Boiling point (°C)
Acetone	23	56.5
Dichloromethane	27.8	39
Hexane	18.43	69
Acetonitrile	19.1	82
DMF	34.4	153
Water	72.7	100

These rules of thumb are largely founded on the theory that vaporisation of the solvent forms a vapor–liquid interface within the MOF, and that the associated surface tension creates capillary stresses on the framework which are strong enough to mechanically destroy the material.^35,38,44,46,50^ This theory of capillary stress-led collapse has been corroborated by the successes of activation procedures avoiding the liquid–gas phase transition through the use of sublimation^51^ or supercritical fluids.^52^ However, to our knowledge these driving forces have yet to be confirmed theoretically and absence of theoretical insight prevents the generation of deeper understanding of key solvent and framework features driving activation-collapse. Furthermore, development of models to describe MOF activation would enable computational prediction of new activation protocols, reducing experimental overhead during materials discovery.

In this study, we computationally investigate MOF activation-collapse to assess the empirical guidelines developed to prevent collapse and generate algorithms for *a priori* prediction of collapse (or lack thereof) during activation. Treating solvent activation as a fluid desorption problem, we apply commonly used grand canonical Monte Carlo (GCMC) techniques to investigate this process. We take advantage of transition matrix Monte Carlo (TMMC)^53^ simulation algorithms to evenly sample the entire potential energy landscape for the activation of each specific MOF–solvent pair. TMMC is a technique widely used to model phase coexistence behaviour of both bulk^54,55^ and confined fluids,^56^ and has been applied to simulate methane in MOFs at subcritical temperatures^57^ and to screen different process conditions.^58^ However, to the best of our knowledge this is the first time that phase behaviour of confined organic solvents is studied using TMMC.

In this paper, we use the Zn_4_O-based IRMOF (isoreticular metal–organic frameworks)^59,60^ family as exemplar materials. These MOFs have the same framework topology but by extending the length of the linkers, *e.g.* using biphenyldicarboxylate (IRMOF-10) instead of benzenedicarboxylate (IRMOF-1), the cavity diameter increases from *ca.* 12 to 17 Å and the window diameter between cavities from *ca.* 8 to 11 Å. Alternatively, the window diameter can be artificially reduced by adding bulky side chains to the linker molecules. We simulate the energetics of desorption as a function of solvent and framework properties, providing clear mechanistic explanation for the phenomenon and hence tools to aid synthetic route planning for experimental researchers.

## Methodology

GCMC simulations were performed using the DLMONTE 2.07 simulation package^61^ (available online at https://gitlab.com/dl_monte), using the dlmontepython package for simulation setup.^62^ Simulation postprocessing was performed in python using the Atomic Simulation Environment (ASE) package,^63^ and visualisation was performed in Paraview.^64^

IRMOF models were taken from the Cambridge Structural Database,^65^ with Mulliken partial charges^66^ calculated using the DFTB + software.^67^ The DFTB3 method^68^ was employed using the 3ob-3-1 parameter set.^69^ Full details of the calculations and sample input files/results are available online at (https://github.com/jrhmanning/TMMC_paper_SI). For atomistic simulations the UFF forcefield^70^ was used for all MOF linker atoms, and DREIDING forcefield parameters^71^ were used for metal node atoms, a combination that has been proven to model adsorption behaviour well.^72^ For sorbate molecules, forcefield parameters were taken from various pre-existing models after validating that TMMC could reproduce both unbiased GCMC simulation results (Fig. S1†) and experimental bulk surface tension values. A complete list of forcefield parameters and references used is provided in Table S2† and comparison against experimental surface tension values is shown in Fig. S2.† All framework and solvent molecules were considered to be atomically rigid during this study.

To describe the solvent–solvent and the solvent–framework interactions, the 12-6 Lennard–Jones potential with Lorentz–Berthelot mixing rules was used. A 12 Å cut-off radius was implemented, after which long-range corrections were applied. Electrostatic contributions were calculated using Ewald summation with the same cut-off distance for real-space interactions. To generate a TMMC free energy bias function across the full range of solvent molecules, simulations in the *μVT* ensemble were performed with the number of solvent molecules constrained to a specific “window”. The resulting transition matrices were then combined by summation. This procedure was repeated twice to smooth out any discontinuities in the bias function, totalling three sweeps across the full range of solvent molecules. In the first sweep, windows of length max(6,*N*_max_/40) were used (where *N*_max_ is the maximum number of solvent molecules per unit cell, as defined during simulation setup), with 1 million GCMC moves attempted per window. In the second sweep, windows of length max(10,*N*_max_/10) were used with 10 million GCMC moves attempted per window. In the final sweep, no window constraints were used with a total of 10 million GCMC moves attempted. Unless stated otherwise, simulations were performed at 298 K and the phase coexistence pressure of the relevant solvent.

**Fig. 1:**
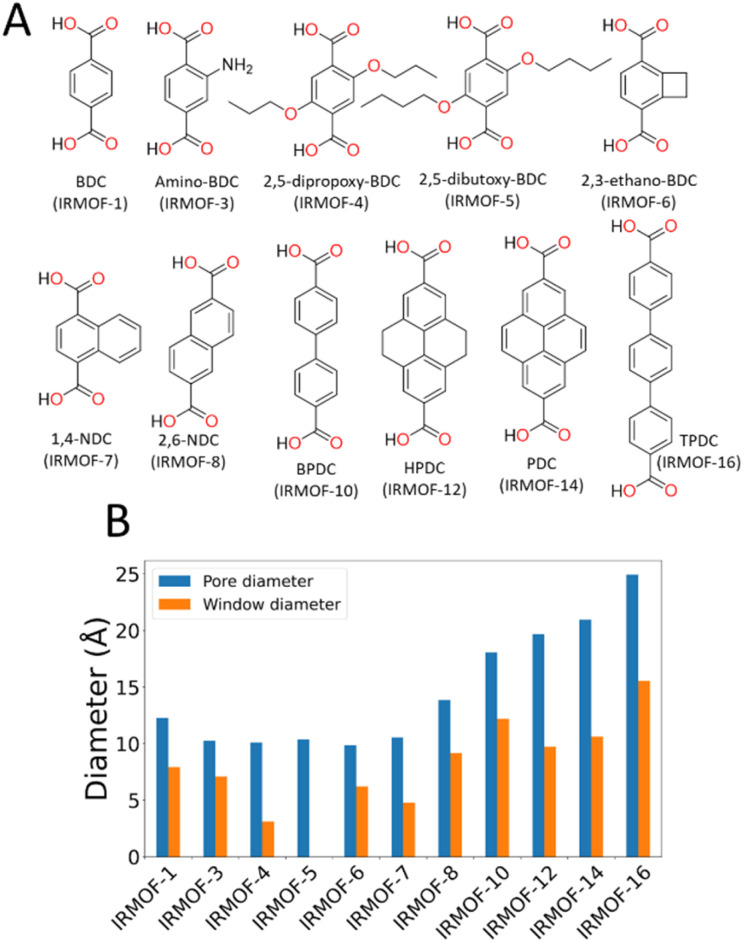
(A) Chemical structures of the linkers of each IRMOF used in this study. BDC = benzene-dicarboxylate, NDC = naphthalene-dicarboxylate, BPDC = bipyridine dicarboxylate, HPDC = tetrahydroxypyrene dicarboxylate, PDC = pyrene dicarboxylate, TPDC = triphenyl dicarboxylate. (B) Comparison of different geometric features of various IRMOF frameworks, displaying the variety of pore (blue) and window (orange) diameters.

To characterise the pore behaviour of MOFs within this study, the pywindow^73^ python package was used to calculate the pore-limiting diameter (PLD) and largest cavity diameter (LCD) of the pores. Linker chemical structure and pore/window diameter are shown in Fig. 1. As framework atomic positions were considered rigid, *n*-propoxy-substituted IRMOF-4 had significantly reduced interconnectivity between pore spaces compared to IRMOF-1, and *n*-pentoxy-substituted IRMOF-5 had no measurable pore interconnectivity. Although no longer representative of the real-world system where the side chains are mobile, simulations considering these frameworks as rigid provide a useful hypothetical example for the case of highly isolated pores with no inter-connectivity.

## Results and discussion

To estimate the energy barrier of solvent activation in MOFs, we used TMMC to calculate the relative free energy of solvents adsorbed within a MOF as a function of solvent density at a constant fugacity and temperature. A free energy (Δ*U*) value of 0 indicates the equilibrium solvent density within the MOF at this particular temperature and fugacity. Multiple solvent density values where Δ*U* = *0* are therefore characteristic of solvent phase coexistence within the framework, and free energy barrier between these density values represents the potential energy of activation (Δ*U*^‡^_act_, where the symbol ‡ denotes the maximum of the free energy barrier) for a specific MOF–solvent pair.

**Fig. 2:**
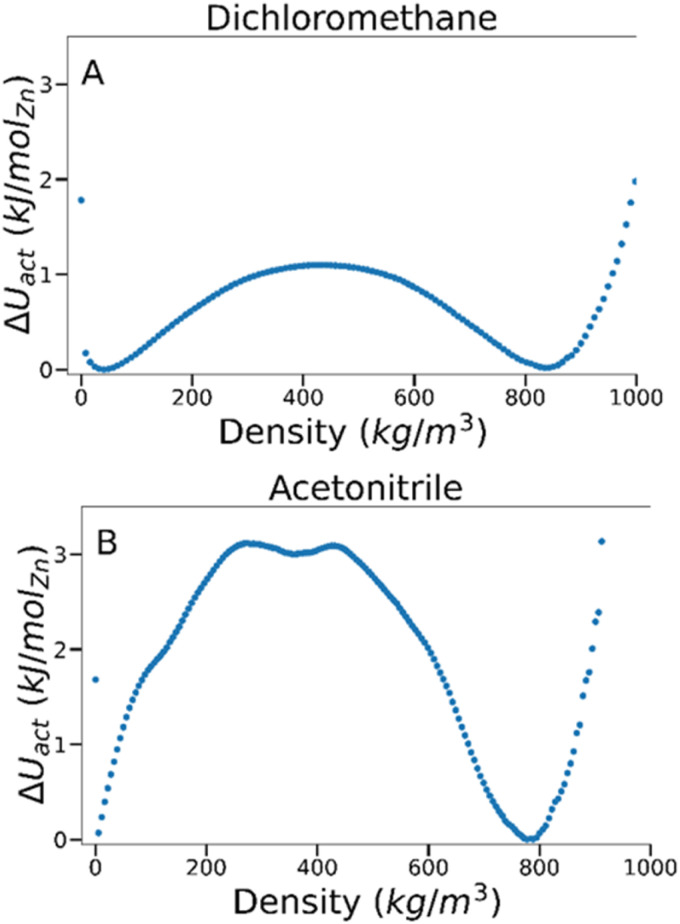
Free energy barriers associated with fluid adsorption/desorption within IRMOF-1 as a function of adsorbed phase density for (A) dichloromethane and (B) acetonitrile at 298 K.

By analysing the magnitude of Δ*U*^‡^_act_ at coexistence for different MOF–solvent pairs, it is possible to investigate the mechanism and energetics of solvent activation. As an example, we present data for dichloromethane and acetonitrile in IRMOF-1 in Fig. 2 which are known to lead to successful activation and activation-collapse, respectively. For both sorbates, phase coexistence is observed between a high density phase at *ca.* 800 kg m^−3^ and a low density phase at <50 kg m^−3^ (at *ca.* 4400 Pa and 490 Pa for dichloromethane and acetonitrile, respectively). The free energy of the phase transition was very distinct, however – Δ*U*^‡^_act_ was significantly lower for dichloromethane (*ca.* 1.1 kJ per mol (Zn)) than for acetonitrile (*ca.* 3.1 kJ per mol (Zn)), suggesting that the key difference between behaviour of the two solvents lies in the nature of the phase change.

To gain a deeper insight into the energetics of phase change, we compared the magnitude of Δ*U*^‡^_act_ against the formation enthalpy of the MOF (Δ*H*_f_). As Δ*H*_f_ represents the energetic cost of entirely decomposing the framework into its constituent terephthalate and Zn_4_O components, it provides an upper limit of framework stability – Δ*U*^‡^_act_ values above Δ*H*_f_ would signify that the MOF is more likely to fully decompose rather than successfully activate. The heat of formation, Δ*H*_f_, of IRMOF-1 has been estimated through experimental^74^ and computational methods^75^ to be −25 and −40 kJ per mol (Zn), respectively. As these values are orders of magnitude higher than the calculated Δ*U*^‡^_act_ in IRMOF-1, collapse caused by acetonitrile removal is not thermodynamically favoured. Instead, some aspect of the phase transition must induce collapse.

**Fig. 3:**
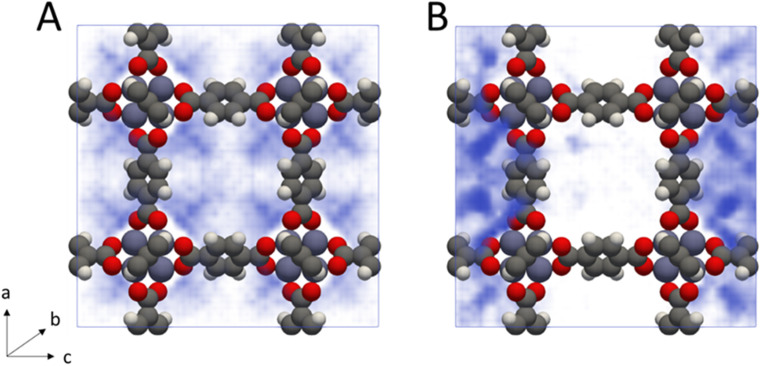
Probability density histograms of (A) dichloromethane and (B) acetonitrile adsorption within IRMOF-1 at densities of 330–410 kg m^−3^ and 200–240 kg m^−3^ respectively (40–50 sorbate molecules per unit cell in each case). Denser regions are coloured in darker blue, while less dense regions are coloured in lighter blue.

From inspection of the acetonitrile free energy curve, several inflection points between the two stable phases were observed (Fig. 2B). The free energy is equal to the fluid surface excess energy,^53,76^ meaning that inflections are indicative of mesophase formation – analogous to bubbles in a bulk fluid.^77^ Further, as this free energy is associated with the formation of an interface, mesophase formation indicates that the energy is not evenly distributed within the MOF – instead the energy is concentrated at specific points within the material. To confirm the presence of acetonitrile mesophases within IRMOF-1 (and the lack of any similar mesophases for dichloromethane), we directly analysed the solvent configurations in the framework during each simulation. By plotting the sorbate probability density distribution within the framework at densities of 330–410 kg m^−3^ and 200–240 kg m^−3^ for dichloromethane and acetonitrile, respectively, mesophase formation can be observed directly (Fig. 3). These sorbate densities correspond to the free energy maxima in Fig. 2, representing the transition state between the two stable phases. In agreement with the free energy profiles, dichloromethane was distributed evenly throughout the framework, with dense clusters surrounding each Zn_4_O secondary building unit and evenly occupying each pore within the framework (Fig. 3A). In contrast, acetonitrile is preferentially located in the larger of the two pores in the MOF (Fig. 3B), forming an interface internally within the framework. Note that simulation in a supercell containing 2 × 2 × 2 unit cells showed the same evenly distributed mesophases demonstrating that the behaviour can be adequately described in the smaller system (animated Fig. S5†).

Phase coexistence within MOF pore spaces has been previously observed in theoretical^78–80^ and experimental^81^ studies of benzene adsorption in IRMOF-1. There, the bimodal pore size of IRMOF-1 – caused by the orientation of the benzene rings in the linker either pointing into or out of the resulting pores – was highlighted. Benzene was shown to preferentially adsorb into the larger of the two pores with a significant energy barrier to move between the pore spaces.^79^ These findings are also in agreement with previous studies showing that phase coexistence within MOFs can exist for any sufficiently subcritical fluid *e.g.* methane at 50 K,^81^ suggesting that the behaviour can be controllably switched by varying the temperature and pressure.

**Fig. 4:**
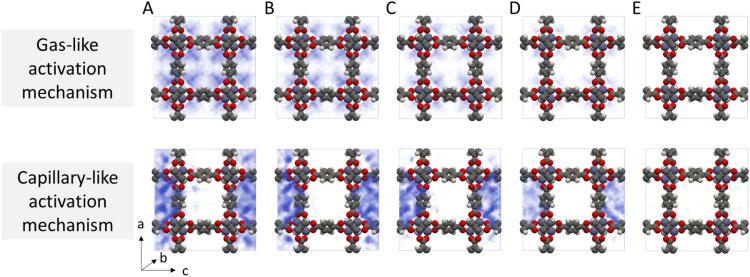
Probability density histograms of (top) dichloromethane and (bottom) acetonitrile in IRMOF-1, demonstrating the distinct solvent clustering behaviour during each activation mechanism. In the gas-like activation mechanism, solvent molecules are extracted equally from all pore spaces, whereas in the capillary-like activation mechanism solvent molecules form concentrated mesophases. IRMOF-1 unit cells contain (A) 40–50, (B) 30–40 (C) 20–30, (D) 10–20, or (E) 0–10 sorbate molecules.

Given the stark difference in adsorption behaviour exhibited by the two different solvents, we identify two potential routes for framework collapse during solvent activation, visualised in Fig. 4. In the first, the solvent shows typical gas-like behaviour, desorbing uniformly from the centre of the pore hence evenly distributing any imparted stress across the framework. In this case collapse only occurs if Δ*U*^‡^_act_ is greater than the thermodynamic stability of the framework. In the second, strong solvent phase cohesion leads to mesophase formation within the MOF pore space at an early stage of desorption. Δ*U*^‡^_act_ is distributed across the resulting interfaces, concentrating the mechanical stress across a relatively small area thereby lowering the barrier to collapse.

While it is clear from Fig. 4 that dichloromethane and acetonitrile desorb from MOFs through different mechanisms, the probability density histograms do not directly link these adsorption mechanisms to collapse. To build this link, we calculated the instantaneous interaction energies acting on each atom of the MOF by the surrounding solvent molecules (Fig. S4†). We found that framework interactions were evenly distributed throughout the framework in the case of gas-like dichloromethane (Fig. S4b†), indicating even capillary stress. Conversely, interactions with capillary-like acetonitrile were highly location-dependent within the unit cell, signifying that some framework atoms experienced far greater stress than their symmetrically equivalent counterparts (Fig. S4c†).

This asymmetric capillary stress cannot be explained by preferential adsorption alone (akin to benzene adsorption into the larger pore space only^81^), as those interactions would remain symmetrically equivalent. Instead, capillary-like sorbates form a single ‘droplet’ within the pore spaces of the MOF, and growth of the single droplet is thermodynamically favoured over both adsorption on the framework or formation of subsequent droplets. Therefore, while the absolute value of Δ*U*^‡^_act_ may not be enough to decompose the IRMOF-1 framework, clustering of sorbate molecules into a single droplet will amplify the local capillary stresses placed on the MOF, triggering breakage of metal–linker coordination bonds which then propagates into framework collapse.

**Fig. 5:**
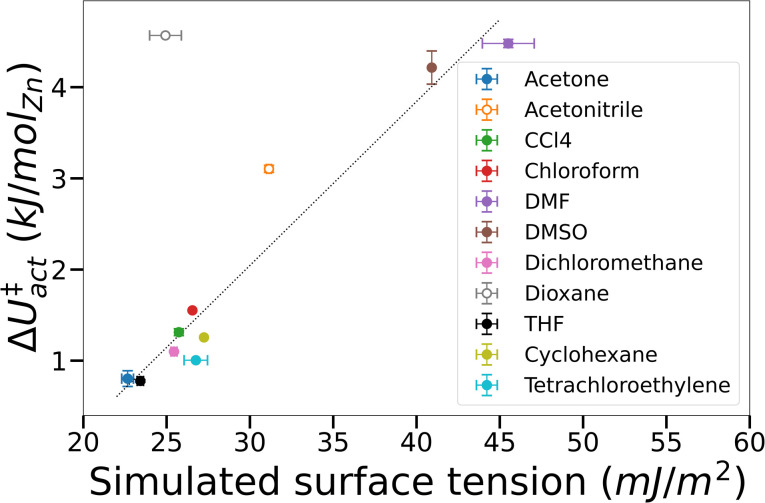
Phase change energy barrier in IRMOF-1 against simulated bulk surface tension. Filled symbols – not mesophase forming, open symbols – mesophase forming. Where error bars are not visible they lie within the symbol size. The straight line was added to guide the eye.

To test the supposition that capillary-like adsorption is a general indicator of activation-collapse for any fluid, we extended our simulations of IRMOF-1 activation to a small library of common activation solvents. These simulations also enable us to test the experimental rule of thumb that activation-collapse behaviour is strongly related to solvent surface tension.^41^ Δ*U*^‡^_act_ can be interpreted as the surface excess energy associated with the interface between a gas and a liquid, which can be transformed to the surface tension by dividing by the interface area.^53,76^ In bulk systems, this is often assumed to be twice the cross-sectional area of the simulation box, as the minimum surface area in a gas–liquid mixture with equal volumes of each phase is a flat slab.^53,76^ Here we apply the same rationale to the fluids confined within the tortuous pore network of a MOF. Although it is impossible to make a similar geometrical assumption to interfaces within a framework, we assume that the interface area is a function solely of the MOF structure *i.e.* is a constant value across all solvents tested. A result of this assumption is that Δ*U*^‡^_act_ = *A*_MOF_·*γ*_bulk_, where *A*_MOF_ is the interface area between the more and less dense solvent phases in the MOF and *γ*_bulk_ is the bulk surface tension of the solvent. These data are plotted in Fig. 5, demonstrating that the assumption is valid for the majority of solvents tested; the assumption only breaks down in the case of solvents where mesophase behaviour was observed in their free energy curves *i.e.* acetonitrile and dioxane (open symbols). This phenomenon is a result of the reduced interface area between the dense and light solvent phases within the MOF (visualised in Fig. 3) – as *A*_MOF_ decreases at constant *γ*_bulk_, Δ*U*^‡^_act_ correspondingly increases.

The additional energy imparted on the framework by mesophase-forming solvents in turn leads to mechanical stress, thereby increasing the likelihood of framework collapse. Inspection of the simulated configurations show that mesophase-forming solvents have weaker solvent–MOF interaction energies compared to gas-like solvents (Fig. S5†), signifying a preference to cluster with other solvent molecules within the pores. In contrast, gas-like solvents preferentially adsorb to the framework ahead of other sorbate molecules, preventing the formation of cohesive phases and interfaces within the MOF. Extrapolating to macroscopic behaviour, our simulations indicate that MOF solvophilicity (*i.e.* the affinity of the solvent towards the MOF) is a greater predictor of successful activation than bulk surface tension. Overall, these findings largely agree with the prevailing theory of activation-collapse – that capillary stresses can lead to physical degradation of the framework^39,41^ – however our results challenge the supposition that bulk surface tension is a good indicator.

To prove that our theory of activation-collapse is general to a variety of materials, we further apply these simulations to other members of the family of IRMOF materials. It is generally accepted^41^ that larger pore diameters will lead to less stable frameworks overall. Accordingly, to independently test the effect of pore size and window diameter on solvent activation, we simulated dichloromethane and acetonitrile removal from IRMOFs with a range of linker lengths and sidechain substitution (linkers shown graphically in Fig. 1A) leading to a range of pore and window diameters (Fig. 1B). These quantities have been widely used as metrics to represent pore size and interconnectivity, respectively.^82^

**Fig. 6:**
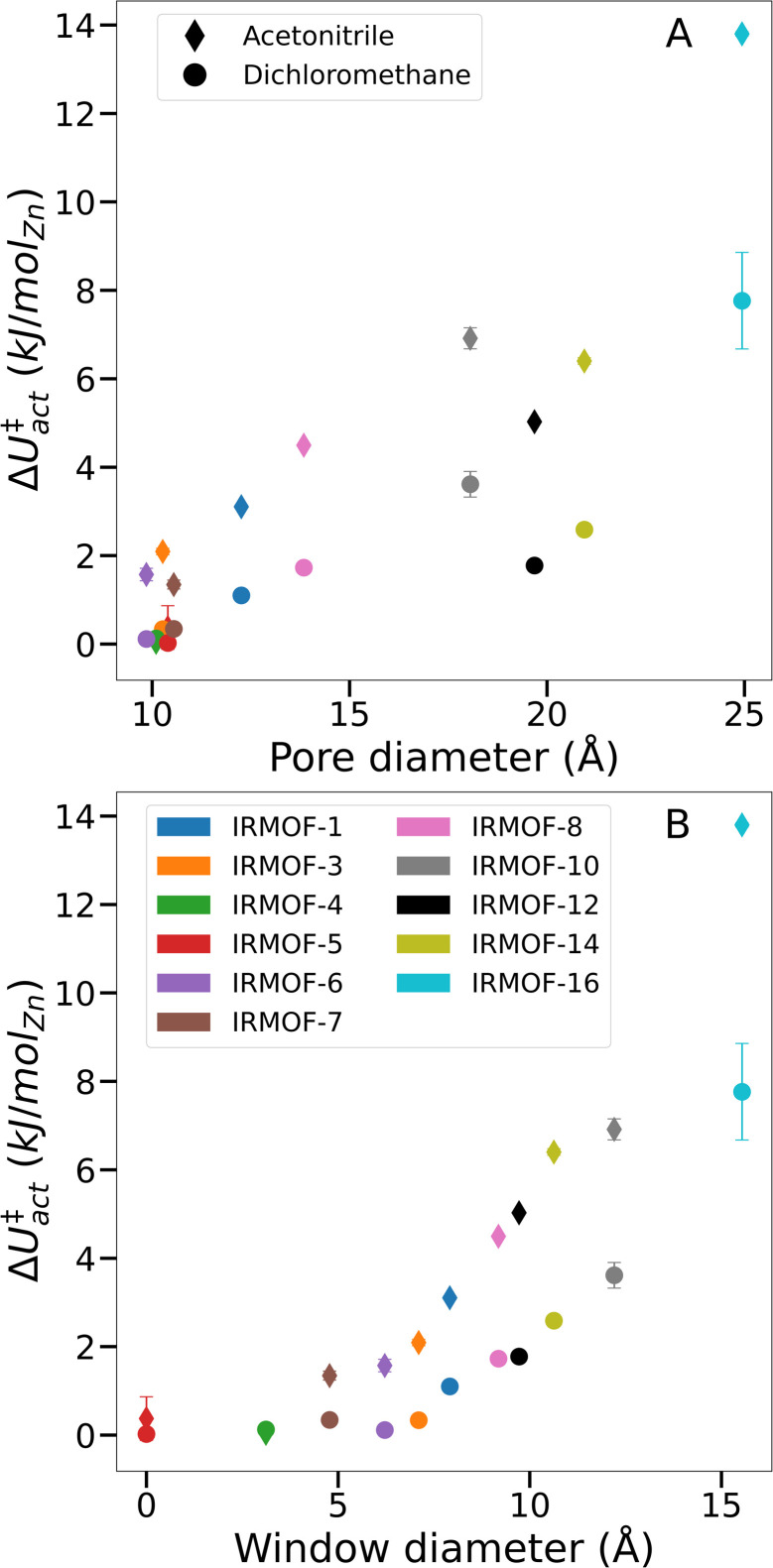
Comparison of Δ*U*^‡^_act_*versus* various system-specific properties. (A) Framework pore diameter, (B) framework window diameter.

Although the bulk surface tension of each solvent is a constant value, Fig. 6 shows that Δ*U*^‡^_act_ varies widely depending on the sorbent material used. Notably, while the relationship between pore diameter and transition energy did not monotonically increase – *i.e.* values for IRMOF-12 and -14 were lower than their smaller-pore equivalents – the relationship between window diameter and transition energy did. The two aspects of pore geometry are clearly correlated. However transition energy appears to be more strongly related to the window diameter and therefore the interconnectivity of the pores rather than the pore size. Similar trends were observed for both dichloromethane and acetonitrile, indicating that greater pore size and connecting window areas will increase the likelihood of activation-collapse. These findings are also in line with the clustering behaviour of acetonitrile in IRMOF-1 described earlier (Fig. 3, S3, and S5†), which shows a tendency to fill an isolated pore before bridging into neighbouring spaces within the MOF.

From simulations in IRMOF-1 (Fig. 5), we propose that solvent mesophase formation reduces the solvent interfacial surface area of the MOF, leading to larger Δ*U*^‡^_act_ and therefore increased likelihood of metal–ligand bond breaking. Given the results from IRMOF-4 and -5 (*i.e.* no measurable energy barrier during solvent desorption, Fig. 6) it appears that there is a minimum pore window diameter required for the formation of solvent mesophases. Further, larger windows (and to a lesser extent larger pore diameters) correspond to both greater Δ*U*^‡^_act_ overall and a greater difference in Δ*U*^‡^_act_ between acetonitrile and dichloromethane for a given framework. As a result, we conclude that solvent interfaces form across the pore window, agreeing with the hypothesis of capillary-like desorption leading to activation-collapse. This new understanding, alongside the insight that mesophase formation is promoted by relatively low MOF-sorbate affinities, can lead to the identification of specific solvents with low likelihood of activation-collapse for a specific MOF. Note, that our findings relate to rigid, 3D MOFs with pores interconnected by windows. Further studies will be necessary *e.g.* in 1D MOFs which do not contain windows but where weak MOF–solvent interaction will still play a key role and flexible MOFs where the framework might adjust while solvent is gradually removed to identify the activation mechanism in these cases. However, the methods developed in this study will enable prediction of specific activation protocols *de novo*, hence designing experimental conditions to avoid collapse, for both newly discovered and existing frameworks.

## Conclusions

Framework collapse upon solvent removal during activation remains a challenge for metal–organic frameworks (MOFs) and a better understanding of the underlying phenomena is needed. In this study, we simulated MOF activation processes for a range of solvents in IRMOF frameworks. Framing activation as a gas desorption problem, we used TMMC to estimate the transition energy of activation (Δ*U*^‡^_act_), revealing differences between good and poor activation solvents and thereby identifying mechanisms for framework collapse. We identified highly contrasting desorption mechanisms between the two groups: good activation solvents behave similarly to a classical gas *i.e.* adsorbing in each pore space approximately uniformly, whereas poor activation solvents form strong mesophases in the pore, resulting in a concentration of stresses across a small number of metal–linker bonds and hence leading to framework collapse. This new analysis enables identification of the activation pathway of a specific MOF–solvent pair, accelerating the development of suitable activation protocols.

By studying various solvents in different frameworks of the IRMOF family, we were able to test experimental rules of thumb governing MOF activation procedures. We corroborated the theory that capillary stress is the primary cause for framework collapse, but rather than resulting from the bulk surface tension of the solvent we identified the formation of mesophases in the pores as the main cause. In turn, this phenomenon was caused by weak solvent–MOF attraction compared to solvent–solvent attraction, predisposing the solvent to cluster within the pore spaces rather than wetting the surface of the framework. In terms of framework properties, we found good agreement with the supposition that larger and more inter-connected pore spaces inherently lead to a higher risk of activation-collapse as Δ*U*^‡^_act_ increases linearly with pore diameter while thermal stability remains largely unchanged.

TMMC simulations therefore represent a powerful tool for predicting the successful activation of MOFs for an arbitrary activation solvent or set of conditions. While this proof-of-concept study focused on a single family of Zn_4_O-MOFs with similar structural features – rigid frameworks with the *pcu* topology – we believe the methods developed here can be applied to any framework material regardless of structural features (*e.g.* mechanical flexibility, pore anisotropy, framework chemistry) when combined with the appropriate simulation parameters and workflows. Using this technique, we envisage computational screening of pressure/temperature conditions during activation, thereby facilitating both MOF discovery and process intensification for scale-up to manufacture. Finally, these methods could be combined with experimental vapour sorption to provide in-depth information about framework–solvent interactions and directly investigate the key factors leading to activation-collapse.

## Conflicts of interest

The Authors declare no competing commercial interests.

## Supplementary Material

TA-011-D3TA04647H-s001

TA-011-D3TA04647H-s002

TA-011-D3TA04647H-s003
