# Supplementary material for: Identifying pathways to metal–organic framework collapse during solvent activation with molecular simulations
Source: J Mater Chem A Mater. 2023 Nov 9;11(47):25929–37. doi: 10.1039/d3ta04647h (PMC10697055; doi:10.1039/d3ta04647h)
Supplement: TA-011-D3TA04647H-s003 [file TA-011-D3TA04647H-s003.pdf]

Electronic Supplementary Material (ESI) for Journal of Materials Chemistry A.
This journal is © The Royal Society of Chemistry 2023

Identifying pathways to metal-organic framework collapse during solvent activation with molecular simulations

Joseph R.H. Manning,*[abc] Gaël Donval,[a] Mat Tolladay,[a] Tom L. Underwood,[d] Stephen C. Parker,[d] and Tina Düren*[a]

Supplementary information

Table S1 – Complete list of solvents used in this study alongside their experimental bulk surface tension (20 °C) and boiling point values. Data are taken from references 1, 2 and 3.

Solvent	Bulk surface tension (mN m^{-1})	Boiling point (°C)
**Acetone**	23	56.5
**Acetonitrile**	19.1	82
**Chloroform**	27.1	61.2
**Cyclohexane**	25.3	80.7
**Dichloromethane**	27.8	39
**1,4-dioxane**	32.5	101.1
**DMF**	34.4	153
**DMSO**	42.9	189
**Tetrachloromethane**	26.8	76.7
**Tetrachloroethylene**	31.7	121.1
**THF**	26.4 (25 °C)	66

Table S2 – Sources of nonbonded interaction parameters used in this study.

Sorbate molecule	Forcefield
**Nitrogen**	TraPPE-small[4]
**Acetone**	TraPPE[5]
**Acetonitrile**	TraPPE[6]
**Chloroform**	OPLS-UA[7]
**Cyclohexane**	TraPPE[8]
**Dichloromethane**	Salas et al.[9]
**1,4-dioxane**	TraPPE[8]
**DMF**	Zeng et al.[10]
**DMSO**	Vahid et al.[11]
**Tetrachloromethane**	Guevara-Carrion et al.[12]
**Tetrachloroethylene**	Goel et al[13]
**THF**	TraPPE[8]

Figure S1 shows the adsorption behaviour of nitrogen in IRMOF-1 at 77 K measured experimentally and simulated from both grand canonical Monte Carlo simulations (labelled unbiased GCMC) and transition matrix Monte Carlo (TMMC). The data from both simulation methods agrees within the error bars thus validating that TMMC can be used to describe adsorption. The shape of the two simulated isotherms is very similar to the experimental isotherm but they are shifted towards lower pressure. We ascribe this to the choice of forcefield used, which has previously been shown to overestimate solid-fluid interaction strengths causing the onset of adsorption at lower pressures than in experiments.[14] Despite this, the experimental and computational data largely agree in other aspects (total quantity adsorbed, isotherm shape), demonstrating that the adsorbed fluid behaviour was correctly modelled by the simulations. Accordingly, only adsorption configurations and energies were analysed in the remainder of this study, and no analysis the specific pressure values of fluid-phase coexistence was carried out.

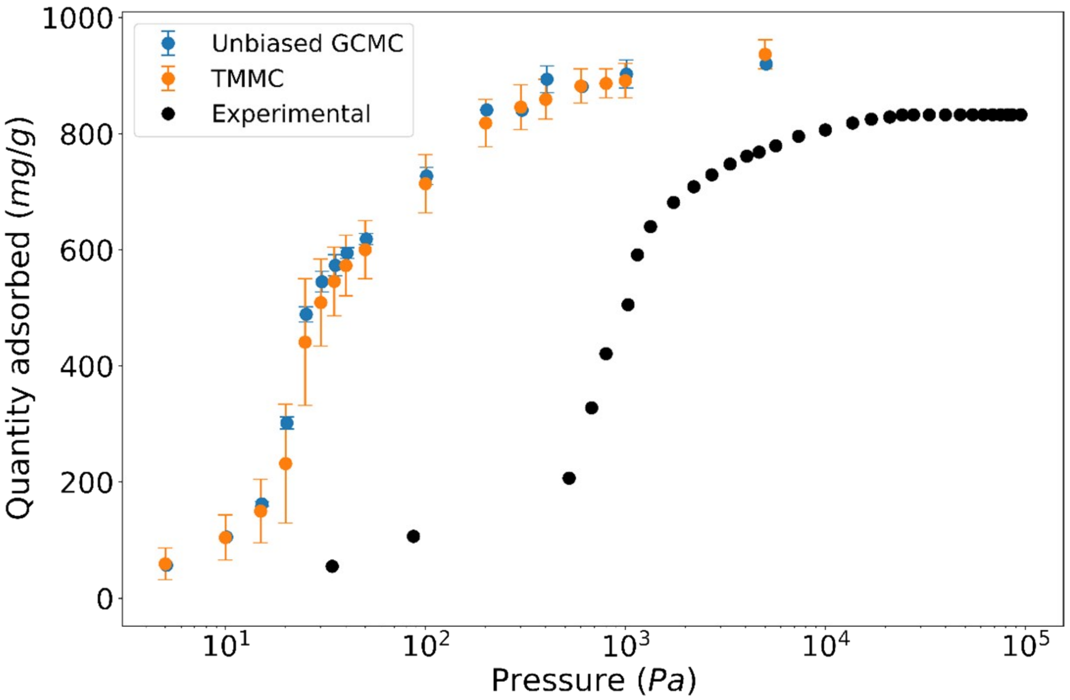

Figure S1 – A comparison of nitrogen adosprtion isotherms in IRMOF-1 at 77 K taken from experimental data (black), unbiased GCMC (blue) and TMMC (orange). Error bars are one standard deviation around the mean for unbiased GCMC, and ± 2 kbT for TMMC. Experimental data traken from reference 15.

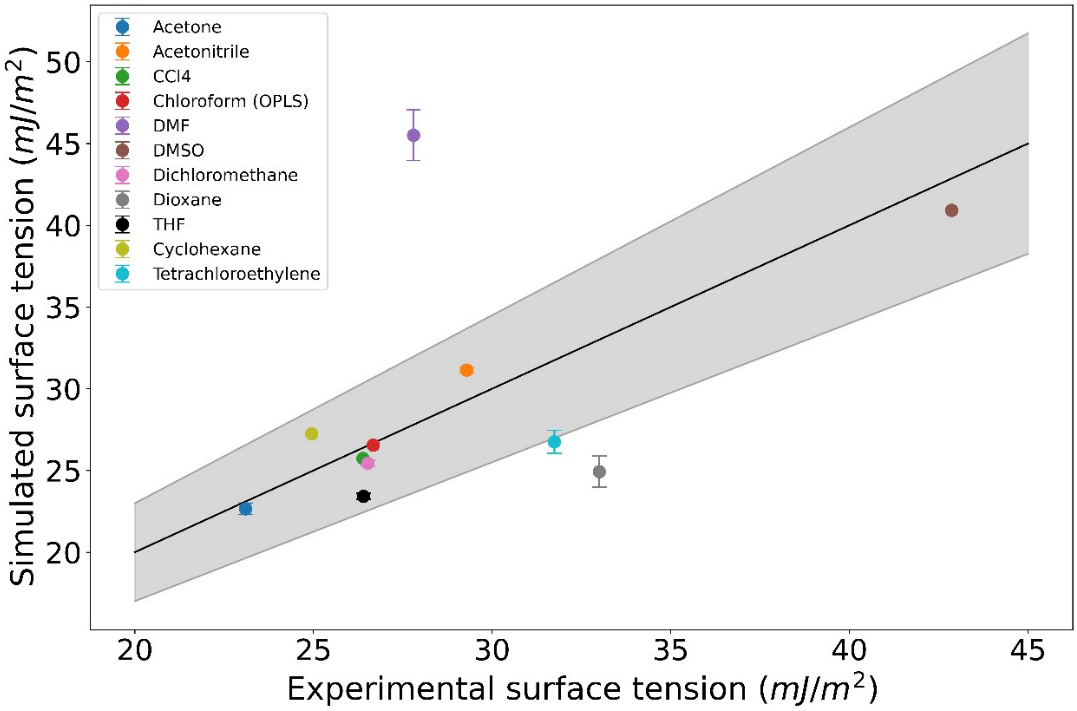

Figure S2 – Comparison between experimental and TMMC-predicted bulk surface tension for solvent compounds used in this study. The black line represents parity between experimental and simulated results, while the shaded are represents 15% uncertainty around the experimental value.

Figure S3 (in external file) – Animation of acetonitrile probability density in IRMOF-1 at densities between 160-200 kgm^{-3} (30-40 acetonitrile molecules per unit cell).

To calculate sorbate phase nonuniformity and clustering within the framework, we calculated instantaneous interaction energies broken down by framework atom. Specifically, we focused on the carbonyl carbon atoms in the linkers, due to their symmetrically equivalent location within all pore spaces, and proximity to the metal-linker bonds which are critical for holding the network together. The distribution of total interaction energies on carbonyl carbon atoms is shown in Figure S4, broken down by their axial orientation within the unit cell (illustrated in Figure S4a). In all cases, the distribution of interaction energies should be approximately identical for all framework atoms due to the symmetrical nature of the crystal unit cell. This is true even in the case of preferential adsorption into different pore spaces within the MOF, as the distribution of pore spaces also remains symmetrically equivalent along the three crystal axes.

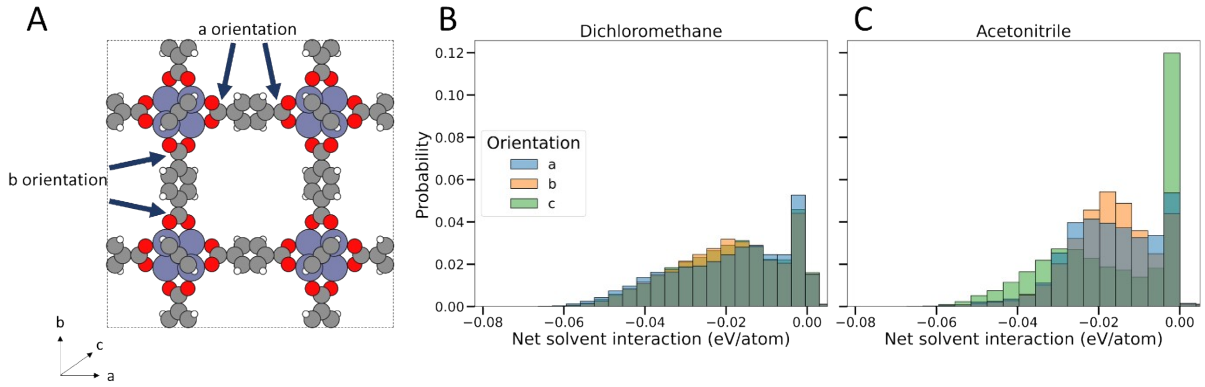

Figure S4 – Breakdown of instantaneous MOF-solvent interaction energies on carbonyl carbons by their orientation within the unit cell. (a) Illustration of the a- *and* b-*axis orientations, along the same projection as in Figure 3 of the main article, (b) histogram of instantaneous energies per carbonyl carbon atom in the case of dichloromethane desorption, (c) histogram of instantaneous energies per carbonyl carbon atom in the case of acetonitrile desorption. Interaction strengths were calculated at sorbate densities of 330-410 kgm^{-3} and 200-240 kgm^{-3} respectively (40-50 sorbate molecules per unit cell in each case).*

The distributions of solvent-carbonyl atom interaction strengths are shown in Figure S4b for dichloromethane and Figure S4c for acetonitrile. Dichloromethane acted as expected within the framework, with approximately identical interactions for all of the carbonyl carbon atoms within the MOF (Figure S4b). Further, this can be visualised in Figures 3, S3, and S5 – sorbate density is periodically distributed throughout the pore space, leading to uniform adsorption stresses in all three crystal planes regardless of dichloromethane's preferential adsorption at sites close to the metal SBUs.

Conversely, histograms of acetonitrile interaction strength show a large difference depending on the linker's axial orientation, indicating heterogeneous phase behaviour within the MOF's pores. This behaviour is clearly asymmetrical, therefore cannot be explained by preferential filling of the larger pore spaces over the smaller (as that would lead to checkerboard-like solvent phases, which remain symmetrical).

Figure S5 (in external file) – Animation of acetonitrile probability density in a 2x2x2 supercell of IRMOF-1 at densities between 160-200 kgm^{-3} (30-40 acetonitrile molecules per unit cell).

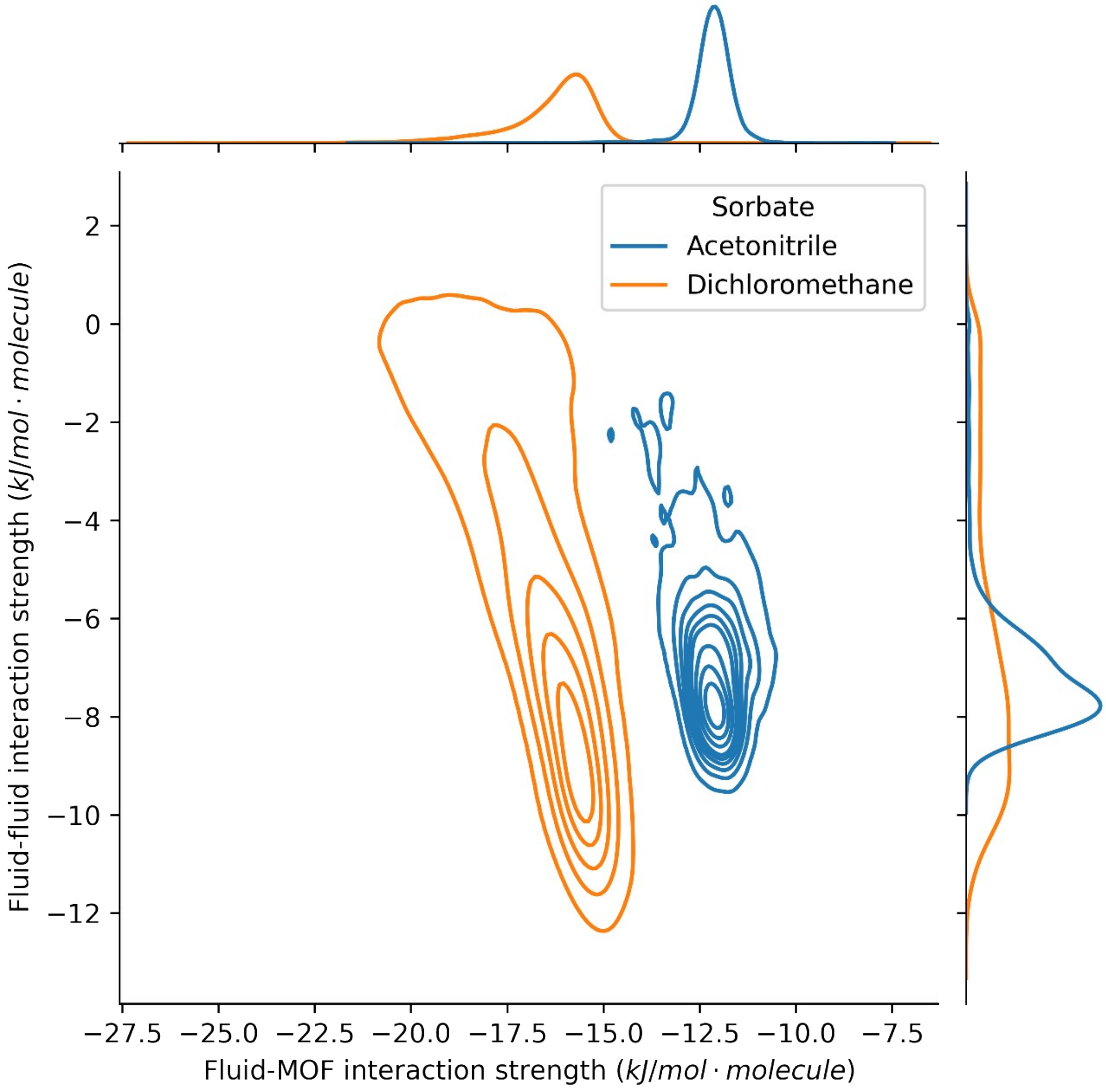

Figure S6 - Comparison of fluid-fluid and fluid-MOF interactions strengths for (blue) acetonitrile and (orange) dichloromethane in IRMOF-1, normalised by number of adsorbed molecules.

References

1 X. Zhang, Z. Chen, X. Liu, S. L. Hanna, X. Wang, R. Taheri-Ledari, A. Maleki, P. Li and O. K. Farha, *Chem. Soc. Rev.*, 2020, **49**, 7406–7427.

2 E. Calvo, A. Penas, M. Pintos, R. Bravo and A. Amigo, *J. Chem. Eng. Data*, 2001, **46**, 692–695.

3 N. A. Lange, *Lange's Handbook of Chemistry*, McGraw-Hill, New York, 14th edn., 1992.

4 J. Stoll, J. Vrabec and H. Hasse, *AIChE J.*, 2003, **49**, 2187–2198.

5 J. M. Stubbs, J. J. Potoff and J. I. Siepmann, *J. Phys. Chem. B*, 2004, **108**, 17596–17605.

6 C. D. Wick, J. M. Stubbs, N. Rai and J. I. Siepmann, *J. Phys. Chem. B*, 2005, **109**, 18974–18982.

7 W. L. Jorgensen, J. M. Briggs and M. Leonor Contreras, *J. Phys. Chem.*, 1990, **94**, 1683–1686.

8 S. J. Keasler, S. M. Charan, C. D. Wick, I. G. Economou and J. I. Siepmann, *J. Phys. Chem. B*, 2012, **116**, 11234–11246.

9 F. J. Salas, G. A. Méndez-Maldonado, E. Núñez-Rojas, G. E. Aguilar-Pineda, H. Domínguez and J. Alejandre, *J. Chem. Theory Comput.*, 2015, **11**, 683–693.

10 A. Zeng, W. Chen, J. Ma and J. Yu, *Trans. Tianjin Univ.*, 2017, **23**, 26–34.

11 A. Vahid and E. J. Maginn, *Phys. Chem. Chem. Phys.*, 2015, **17**, 7449–7462.

12 G. Guevara-Carrion, T. Janzen, Y. M. Muñoz-Muñoz and J. Vrabec, *J. Chem. Phys.*, 2016, **144**, 1–23.

13 H. Goel and N. Rai, *ACS Omega*, 2018, **3**, 3646–3654.

14 D. Fairen-Jimenez, R. Galvelis, A. Torrisi, A. D. Gellan, M. T. Wharmby, P. A. Wright, C. Mellot-Draznieks and T. Düren, *Dalt. Trans.*, 2012, **41**, 10752.

15 M. Eddaoudi, J. Kim, N. Rosi, D. Vodak, J. Wachter, M. O'Keeffe and O. M. Yaghi, *Science*, 2002, **295**, 469–472.
